# Supplementary material for: Homotypic interactions between SLAMF1 receptors on innate T cells and neutrophils regulate killing of fungi
Source: PLoS Pathog. 2026 May 28;22(5):e1014282. doi: 10.1371/journal.ppat.1014282 (PMC13229365; doi:10.1371/journal.ppat.1014282)
Supplement: S1 Text — (DOCX) [file ppat.1014282.s005.docx]

**MATERIALS AND METHODS**

*Fungi*

*Blastomyces dermatitidis* strains used were wild-type ATCC strain 26199 and DsRed26199[1]. The *fungus* was grown as yeast on Middlebrook 7H10 agar with oleic acid-albumin complex (Sigma) at 39 ̊C.

*Mice*

SLAMF1 knockout mice were generated and obtained from Dr. Cox Terhorst at the Beth Israel Deaconess Medical Center at the Harvard Medical School in Boston[2]. C57BL/6 mice were obtained from Jackson Laboratory and bred at our facility. Heterozygous SLAMF1^fl/+^ were obtained from Shanghai Model Organisms and bred to homozygosity. Homozygous SLAMF1^fl/fl^ were crossed with cell specific cre mice (obtained from The Jackson Laboratory to generate conditional knockout mice. Cre mice were B6.FVB-Tg(EIIa-cre expressed in embryonic cells) C5379Lmgd/J (stock #003724), B6(129X1)-Tg(Cd4 cre/ERT2 expressed by CD4^+^ T cells)11Gnri/J (stock #22356), B6.129S-Tcrdtm1.1(cre/ERT2 expressed by TCRγδ^+^ T cells)Zhu/J (stock #031679) and C57BL/6-Ccr2em1(icre/ERT2 expressed by monocytes) Peng/J (stock #035229). Conditional CCR2-Cre^+^ x SLAMF1*^fl/fl^* mice were generated using *CCR2-CreER-GFP* mice (stock #035229) that express *cre-ER^T2^* and *EGFP* directed from the endogenous chemokine (C-C motif) receptor 2 (*Ccr2*) promoter.

*Generation of conditional SLAMF1 knockout mice*

To generate conditional SLAMF1 knockout mice we first bred commercial SLAMF1^fl/+^ mice to homozygosity (**S3A Fig**). To validate homozygous SLAMF1^fl/fl^ mice we crossed them with EIIa-cre mice[3], which carry a cre transgene under the control of the adenovirus EIIa promoter that targets expression of Cre recombinase to the early mouse embryo, and are useful for germ line deletion of *loxP*-flanked genes. If EIIa cre x SLAMF1^fl/fl^ mice ablate SLAMF1 at the germline level, they should lack SLAMF1 expression throughout the body and exhibit a similar resistance phenotype to *Bd* infection as seen with the original full body SLAMF1 knockout mice (**Fig 1A+1B**). SLAM1 staining and FACS analysis showed that CD4^+^ T cells, TCRγδ^+^ T cells and monocytes from EIIa cre x SLAMF1^fl/fl^ mice lacked SLAMF1 expression similar to full body SLAMF1 knockout mice (**S3B Fig**) and exhibited accelerated death and increased lung CFU upon infection with *Bd* (**S3C+S3D Fig**). These results indicated that EIIa cre x SLAMF1^fl/fl^ mice phenocopied SLAMF1 knockout mice.

Lung CFU and survival

Mice were challenged with 2x10^4^  *B. dermatitidis* yeast. When mice became moribund lungs were harvested and plated for lung CFU or euthanized for generating a survival curve.

*Fungal infection and Lung processing*

Mice were challenged intratracheally with 10^5^ Uvitex stained DsRed 26199 yeast. Lungs were harvested in wash buffer and dissociated in Miltenyi MACs tubes, then digested with 5ml collagenase D solution (collagenase buffer (10mM HEPES, 5mM KCl, 2.1 mM MgCl_2_, 1.8 mM CaCl_2_, 241 mM NaCl/ml) with 1mg/ml Collagenase D, 1μg/mL DNAse) for 30 min at 37C. Digestion was stopped with 100μl of 0.5M EDTA (Invitrogen 15575-038). For tracking the association and killing of yeast by phagocytes, the erythrocytes were lysed and lung cells stained for myeloid markers as previously described [4]. Alternatively, for the isolation of lung leukocytes, digested lungs were resuspended in 5 ml of 40% Percoll in wash buffer; 3 ml of 66% Percoll in PBS was underlaid (17-0891-01; GE Healthcare). Samples were spun for 20 min at 550 RCF at 22C. Lymphocytes were then harvested from the buffy coat layer and resuspended in desired media.

*Isolation of target cells from the lung and bone marrow*

CD4^+^ and TCRγδ^+^ T cells were isolated from the lungs of infected mice (16 hours post-challenge) following lung processing protocol referenced above to harvest leukocytes, then according to the manufacture’s isolation protocols (Miltenyi Isolation kits (CD4+T cells; Cat#130-104-454 and TCRγδ; Cat# 130-092-125). Monocytes were isolated from the bone marrow following the manufacturer’s protocol (Monocyte: Cat# 130-100-629). Cell populations before and after isolation and negative fractions were stained for SLAMF1 and the purity assayed by FACS.

*In vitro killing assay*

Neutrophils were used as effector cells for killing the yeast and harvested from bone marrow of wild type C57BL/6 mice. Bone marrow was flushed with PBS and aspirated to disaggregate large bone marrow pieces from hind legs and filtered through a 70 mM cell strainer (Fisher Cat# 22362548). Suspension was pelleted then resuspended in 6 mL of 0.2% NaCl to lyse red blood cells (RBCs). After 10 seconds, osmolarity was restored with the addition of 14 ml of 1.2% NaCl. Cell suspension was strained with a 70 mM cell strainer and pelleted. Pellet was resuspended in 5 ml of wash buffer and pipetted on top in a tube of 5mL 62% Percoll in PBS with 2 ml of 83% Percoll in wash buffer underlaid. Samples were spun for 30 min at 1000 RCF at 22C. Neutrophils were then harvested from buffy coat layer between 62% and 83% Percoll, washed with wash buffer, and resuspended to 4.5x10^6^/ml in complete RPMI (cRPMI, RPMI with 10% FBS, 1% penicillin and streptomycin). Coculture assays were set up in an untreated 96 well plate (Avantor #10861-561) as follows: 50,000 yeast, 450,000 neutrophils or 120,000 monocytes (as phagocyte effector cells), and additional cell types designed to interact with phagocytes ranging as follows: 4.5x10^5^ to 1x10^6^ for CD4^+^ T, 1x10^5^ to 1.5x10^5^ for TCRγδ^+^ T cells, and 1x10^5^ to 2x10^5^ for monocytes. For yeast only or yeast and neutrophil groups, wells were filled to a final volume of 300 μl with cRPMI. Samples were incubated for 16 hours before plating for CFU.

*In vitro transwell assay*

Neutrophils were isolated as described in the *in vitro* killing assay. Neutrophils were concentrated to 4.5x10^6^/ml and 26199 yeast concentrated to 0.5x10^6^/ml. 50μl of yeast, neutrophils, and target cell types were placed in the upper well of a 96 transwell system with a 0.4mm polycarbonate membrane (Corning 3381) to allow for homotypic interactions. For yeast only or yeast and neutrophil groups, the upper well was filled to a volume of 150 μl with cRPMI. For positive control group, human recombinant IFN-g (R&D Systems 285-IF-100) in cRPMI was added to the top well for a final concentration of 150U/ml.[5] The bottom well was seeded with 100 μl of yeast and effector neutrophils. For the yeast only control group, cRPMI was added for a final volume of 200 μl. The transwell assay was incubated at 37C for 16 hours. Yeast from the bottom well were plated to determine CFU.

*In vivo killing assay*

To assess the ability of phagocytes to kill yeast *in vivo*, we employed Ds-Red 26199 yeast that loses red fluorescence in dead yeast as described [1,6]. Briefly, yeast were concentrated to 5x10^6^/ml and 0.5 ml was stained with 10μl of 1mg/ml Uvitex in the dark at 22C. After washing the yeast, the mice were challenged with 10^5^ Uvitex stained Ds-Red yeast. FMO controls were included by challenging mice with unstained 26199 yeast, Ds-Red yeast, and Uvitex stained 26199 yeast. Lungs were harvested 16 hours after challenge in wash buffer (RPMI with 1% FBS, 1% penicillin and streptomycin) and dissociated in Miltenyi MACs tubes and digested with 5ml collagenase D solution (collagenase buffer with 1mg/ml Collagenase D, 1ug/ml DNAse) for 25 min at 37C. Digestion was stopped with 100 μl of 0.5M EDTA (Invitrogen 15575-038) and RBCs were lysed for 4 min with 5ml ACK lysis buffer (Gibco A10492-01) then diluted with 20ml of wash buffer. Cells were washed and resuspended to a concentration of 2x10^7^/ml for staining. Samples were stained with LIVE/DEAD Fixable Near-IR Dead Cell Stain Kit (L34975; Invitrogen) and Fc block for 10 min at room temperature. Samples were surface stained at 4C for 30 minutes, following by fixation with 2% PFA (16% PFA diluted with ddH_2_O; VWR Cat# 28908). Panel included a dump channel to reduce non-specific staining. The antibody cocktail consisted of Siglec F BB515 (E50-2440; Cat # 564514; BD), Ly6G PerCP-Cy5.5 (1A8; Cat #127616; Biolegend), CD11c PE-Cy7 (N418; Cat# 117317; Biolegend), Ly6C BV785 (HK1.4; Cat# 128041; Biolegend), MHCII A647 (M5/114.15.2; Cat# 107618; Biolegend), TCRβ BUV395 (H57-597; Cat# 569248; BD), B220 BUV395 (RA3-6B2; Cat#563793; BD), Nk1.1 BUV395 (PK136; Cat# 564144; BD), CD11b BV737 (M1/70; Cat# 612800; BD).

*ROS and NO staining*

Sample aliquots from *in vivo* killing assay were taken at staining step to be stained for ROS and NO. Samples were placed in a 96 well plate. For ROS staining the samples were stained with 10μg/ml DHR-123 (Invitrogen D23806) and incubated for 3 hours at 37C then surface stained following *in vivo* killing assay protocol. For the NO staining the samples were stained with 10 μM DAF-FM diacetate (Invitrogen D23844) and incubated for 10 to 20 minutes at 37C then washed with PBS and incubated for another 15 minutes. Cells were surfaced stained following *in vivo* killing assay protocol.

*Ex vivo SLAMF1 staining - Lungs*

Leukocytes from the percoll gradient were stained with LIVE/DEAD Fixable Near-IR Dead Cell Stain Kit (L34975; Invitrogen) and Fc block for 10 min at room temperature. Samples were surface stained at 4C for 30 minutes, following by fixation with 2% PFA (16% PFA diluted with ddH_2_O; VWR Cat# 28908). The antibody staining panel consisted of SLAMF1 FITC (mShad150; Cat# 11-1502-82; Invitrogen) or BV785 (TC15-12F12.2; Cat# 115937; Biolegend), TCRγδ PerCP-Cy5.5 (GL3; Cat# 118118; Biolegend), Siglec F PerCP-Cy5.5 (E50-2440; Cat# 565526; BD), IL33Ra PE (DIH9; Cat# 145304; Biolegend), CD64 PE (X54-5/7.1; Cat# 139304; Biolegend), CD19 PE Dazzle (6D5; Cat# 115554; Biolegend), TCRβ PE-Cy7 (H57-597; Cat# 109222; Biolegend), CD11c PE-Cy7 (N418; Cat# 117317; Biolegend), NK-1.1 BV421 (PK136; Cat# 108741; Biolegend), CD103 eFluor450 (2E7; Cat# 48-1031-82; Invitrogen), Ly6C BV510 (HK1.4; Cat# 128033; Biolegend), CD8a BV650 (53-6.7; Cat# 100742; Biolegend), CD11b BV650 (M1/70; Cat# 101239; Biolegend), CD90.2 BV785 (30-H12; Cat# 105331; Biolegend) or BUV737 (53-2.1; Cat# 741701; BD), CD196 AF647 (140706; Cat# 557976; BD), MHCII AF700 (M5/114.15.2; Cat# 107622; Biolegend), CD4 BUV395 (GK1.5; Cat# 563790; BD), Ly6G BUV395 (1A8; Cat# 563978; BD).

*Surface and intracellular staining of SLAMF1 of neutrophils from the bone marrow*

Bone marrow was harvested as described above and stained with the LIVE/DEAD Fixable Near-IR Dead Cell Stain Kit and Fc block for 10 min at room temperature. Samples were surface stained at 4C for 30 minutes using the SLAMF1 antibody panel as described above, washed with FACS buffer, then incubated in 100 μl of Cytofix/Cytoperm (BD Cat# 554714) at 4C for 20 minutes. Samples were washed twice with Perm/Wash buffer (BD Cat# 554714) and stained intracellularly with anti-CD150 BV-785 (TC15-12F12.2; Cat# 115937; Biolegend), washed again with Perm/Wash buffer, then fixated with 2% PFA (16% PFA diluted with ddH_2_O; VWR Cat# 28908). We also stained neutrophils that were exposed to yeast overnight. 4.5x10^5^ neutrophils and 5x10^4^ yeast in cRMPI were incubated for 16 hours in a 96 well plate prior to staining.

*Flow Cytometry*

Samples were acquired on an LSR Fortessa and Cytek Aurora Spectral Cytometer, at the University of Wisconsin Carbone Cancer Center Flow Lab.

*Statistics*

All statistics were calculated in Prism 10 for Mac OS X, version 6.1. Differences in fungal burden (expressed as CFU) between two groups were analyzed by the Mann-Whitney U test for ranking data. In some instances, 1-way ANOVA was used when comparing multiple groups, and when a result was significant, a Tukey’s or Dunnett’s post hoc test was used to adjust for multiple comparisons. For comparison of fungal burden among three or more groups of mice, the Kruskal-Wallis test, a nonparametric ranking method, was used. Survival data were examined by the Kaplan-Meier test using log rank analysis to compare survival plots as reported previously (6). A p value of <0.05 was considered statistically significant. Comparisons in many experiments yielded p values at or below the value of p = 0.05, however we consistently used only one asterisk throughout to denote any statistically significant difference regardless of the exact value below 0.05.

**Supplemental References**

1. Sterkel AK, Lorenzini JL, Fites JS, Subramanian Vignesh K, Sullivan TD, et al. (2016) Fungal Mimicry of a Mammalian Aminopeptidase Disables Innate Immunity and Promotes Pathogenicity. Cell Host Microbe 19: 361-374.

2. Wang H, LeBert V, Hung CY, Galles K, Saijo S, et al. (2014) C-type lectin receptors differentially induce th17 cells and vaccine immunity to the endemic mycosis of North America. The Journal of Immunology 192: 1107-1119.

3. Lakso M, Pichel JG, Gorman JR, Sauer B, Okamoto Y, et al. (1996) Efficient in vivo manipulation of mouse genomic sequences at the zygote stage. Proc Natl Acad Sci U S A 93: 5860-5865.

4. Sterkel AK, Mettelman R, Wuthrich M, Klein BS (2015) The unappreciated intracellular lifestyle of Blastomyces dermatitidis. J Immunol 194: 1796-1805.

5. Marchi LF, Sesti-Costa R, Ignacchiti MDC, Chedraoui-Silva S, Mantovani B (2014) In vitro activation of mouse neutrophils by recombinant human interferon-gamma: increased phagocytosis and release of reactive oxygen species and pro-inflammatory cytokines. International immunopharmacology 18: 228-235.

6. Wang H, Lee TJ, Fites SJ, Merkhofer R, Zarnowski R, et al. (2017) Ligation of Dectin-2 with a novel microbial ligand promotes adjuvant activity for vaccination. PLoS Pathog 13: e1006568.
