# Supplementary material for: Homotypic interactions between SLAMF1 receptors on innate T cells and neutrophils regulate killing of fungi
Source: PLoS Pathog. 2026 May 28;22(5):e1014282. doi: 10.1371/journal.ppat.1014282 (PMC13229365; doi:10.1371/journal.ppat.1014282)
Supplement: Attachment — Submitted filename: Rebuttal letter final .docx [file ppat.1014282.s008.docx]

May 12, 2026

Editor

*PlosPathogens*

**RE: Revision of manuscript PPATHOGENS-D-26-00450**
Homotypic interactions between SLAMF1 receptors on innate T cells and neutrophils regulate killing of fungi

Dear Editor:

We were pleased with the favorable disposition and appreciate the chance to revise our paper. We have generated new data that we include in the revised the paper in response to the reviewers’ and your requests. We provide a point-by-point response to the concerns raised. In the revision, we highlight in yellow the text with new data or discussion requested. We also provide below a bulleted summary of new results requested by reviewers that now appear either in the revision or the rebuttal letter.

- Requested leukocyte recruitment data to the lung in the absence and presence of SLAMF1 (see letter below)
- Requested data on augmented SLAMF1 mediated killing of yeast by monocytes (see revised manuscript)

**Reviewer #1:**

**Query 1)** The presented data convincingly show the role of SLAMF1 in phagocytic fungal killing. Do the authors see any role for SLAMF1 in leukocyte accumulation/recruitment

**Response:** We appreciate the reviewer’s question and analyzed leukocyte recruitment to the lung in the absence and presence of SLAM1. At 16 hours post-infection, when the burden of lung infection was comparable between SLAMF-sufficient and -deficient mice we stained lung cells with a panel of multiplexing antibodies to identify 17 pulmonary leukocyte subsets with 12 color flow cytometry. We show these data in the figure below. We found that CD4^+^ T cells, TCRγδ^+^ T cells, monocytes and neutrophils increased 2-4 fold in the lungs of infected wild type mice compared to naïve controls. In contrast, the accumulation of these leukocyte populations (particularly monocytes and neutrophils) was reduced in SLAMF1-knockout mice. Reduced influx of SLAMF1-deficient monocytes has also been reported in a model of colitis in mice (*Gastroenterology*. 2012 December ; 143(6): 1544–1554). Similarly, migration of myeloid cells (dendritic cells and macrophages) was reduced in a model of skin sensitization to induce peritonitis (*PLoS ONE* 10(3): e0121968). These results are consistent with the idea that reduced influx of monocytes and neutrophils in SLAMF1-knockout mice could contribute to increased lung CFU compared to wild type control mice. However, these results are preliminary and outside the scope of this short report that describes how homotypic SLAMF1interactions with innate T cells augment phagocyte function (killing).

**Legend:** SLAMF1-sufficient and -deficient mice were infected with 20,000 *B. dermatitidis* yeast and lungs harvested at 16 hours post-infection. Leukocytes were stained with multiplexing antibodies to identify 17 pulmonary leukocyte subsets by FACS analysis. The n-fold change in cell numbers is shown and was caculated by comparing the geometric means of the corresponding cell population before and after infection. *, p < 0.05 using Anova test.

**Query 2)** The authors focus their attention on neutrophils as there were ~4-fold higher number of neutrophils associated with the fungus than monocytes. While this is reasonable, as monocytes also participate in fungal killing and harbor SLAMF1, does a similar homotypic interaction-mediated enhancement of killing is observed for monocytes too?

**Response:** The reviewer raises an important question. We performed an *in vitro* coculture assay with CD4^+^ T cells and monocytes as the effector cells similar to how we investigated fungal killing by neutrophils. We found that the addition of CD4+ T cells augmented killing of the yeast by monocytes. Hence, as the reviewer suspected, homotypic SLAMF1interactions mediate augmented fungal killing by monocytes as well. We include these new data in **Fig. 2F** and discuss them in the results/discussion on page 6 lines 91 and 92 of the revised manuscript.

**Query 3)** Does the absence of SLAMF1 on CD4 T cells lead to impaired host survival?

**Response:** Our published experience is that the burden of lung infection mirrors survival. We have reported this relationship in a number of publications (*Cell Host & Microbe* 2015, 17, 1–14; J. *Clin. Invest*. 2000, 106:1381–1389; *mBio* 2021, 12; *PLoS Pathogens* 2017, 13 (8): e1006568; *The Journal of Immunology*,2014, 192: 1107–1119; The *Journal of Immunology*, 2022, 208: 1417 1423; and *PLoS Pathog 2016, 12* (8): e1005787. We found that the relationship between lung burden and survival also holds true in the current manuscript when we analyzed lung CFU and survival of infected wild type and SLAMF1 knockout mice as shown in **Fig. 1A+B**. Thus, we would predict that CD4-Cre+ x SLAMF1*^fl/fl^* mice would die in an accelerated fashion since they have a significantly higher lung burden compared to CD4-Cre- x SLAMF1*^fl/fl^* controls. Unfortuntatley, we did not have enough of these mice to perform the survival experiment in sufficient time for the revision.

Minor Issues: Editorial and Data Presentation Modifications
Please use this section for editorial suggestions as well as relatively minor modifications of existing data that would enhance clarity.

**Reviewer #1:**

**Query 1)** In Figure 1C, the labels hide the flow events on the plot. Please modify the label position so that the events are visible.

**Response:** We have modified the figure. We reduced the size of the black box and the writing reporting the percentages of dead yeast slightly so it doesn’t cover the dots from the dot plot anymore. Please note that the box is now below the threshold of events that we are showing.

**Query 2)** The mouse fungal challenge method should include additional details on route of administration, and how lungs were homogenized.

**Response:** We added that information requested in S1 text that contains the Methods on pages2+3, lines 41-52.

**Query 3)** For the in vitro killing assay, please specify the effector-to-target ratios used for the ease of interpretation.

**Response:** We used 50,000 yeast and 450,000 neutrophils and CD4+ T cells ranging from 100,000 to 450,000 cells. We have added that information to S1 text that contains the Methods on page 4 lines 76 and 77.

We hope that the Editors agree that the paper is stronger and is now suitable for publication.

Sincerely yours,

Marcel Wuethrich, PhD
